# *Tinea nigra* Presenting Speckled or “Salt and Pepper” Pattern

**DOI:** 10.4269/ajtmh.13-0394

**Published:** 2014-06-04

**Authors:** André Luiz Rossetto, Rosana Cé Bella Cruz, Vidal Haddad Junior

**Affiliations:** Department of Dermatology, University of Vale do Itajaí (Univali), Itajaí, SC, Brazil; Department of Pharmaceuticals-Biochemistry, University of Vale do Itajaí (Univali), Itajaí, SC, Brazil; Department of Dermatology, Botucatu School of Medical, Universidade Estadual Paulista “Júlio de Mesquita Filho”–São Paulo, State University (FMBUNESP), Vital Brazil Hospital, Butantan Institute, São Paulo, SP, Brazil

## Abstract

A 7-year-old Caucasian female resident of the southern coast of Brazil presented dark spots on the left palm that converged to a unique macule with speckled pattern at about 1 month. The mycological exam and the fungi culture were typical of *Hortaea werneckii*, the agent of the superficial mycosis *Tinea nigra*. The patient received butenafine hydrochloride 1% for 30 days, resulting in a complete remission of the lesion. At a follow-up visit 12 months after treatment, there was no lesion recurrence. We describe a form of rare geographical *Tinea nigra* with a speckled pattern. The “salt and pepper” aspect should be taken into consideration when the mycosis was suspected.

A 7-year-old Caucasian female child, who was a resident of Itapema town on the northern coast of Santa Catarina State, Brazil, presented dark spots on the left palm about 1 month before the exam; they converged to a unique macule with speckled pattern, with a 1.5 × 2.0-cm diameter and geographic shape (Figure 1).

**Figure 1.**
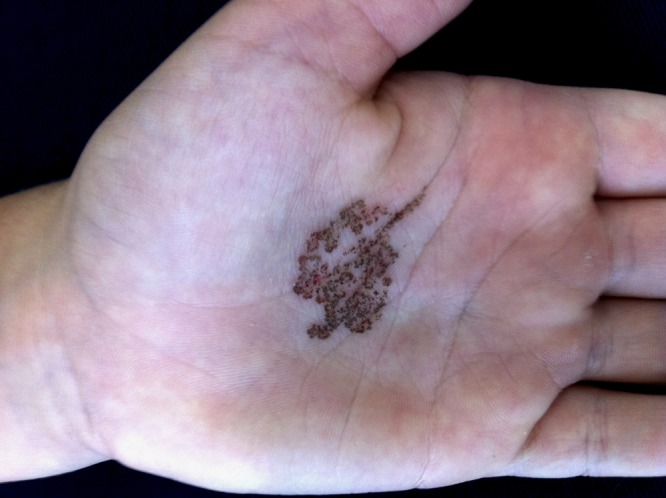
Black macule with geographic shape and speckled pattern on the left palm of the Caucasian patient.

The mycological exam revealed septate, dematiaceous hyphae, and presence of yeast-like cells with spores. The fungi culture in Sabouraud's agar showed a moist, shiny, rough, and black colony. The fungi in the culture were identified as *Hortaea werneckii* (Figure 2).

**Figure 2.**
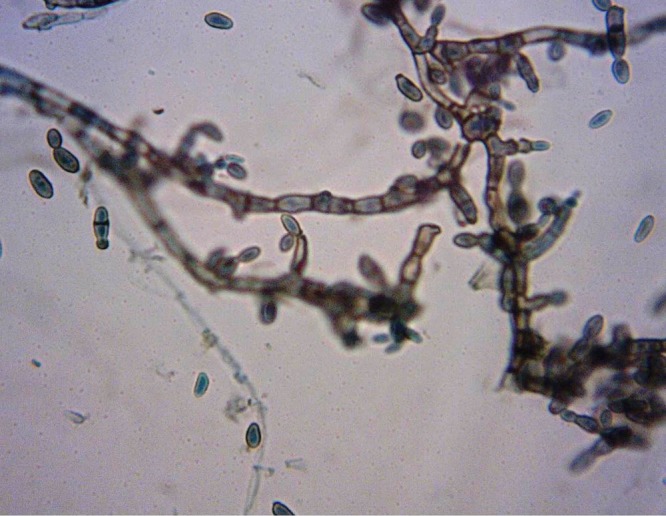
Presence of annelloconidia with pigmented filaments (Magnification: 400×).

The topical treatment with butenafine hydrochloride 1% for 4 weeks caused complete remission of lesions, with no recurrence during follow-up for 1 year.

Reports of *Tinea nigra* have been rare since the first publication by Cerqueira in 1916.^1^,^2^ This cosmopolitan dermatomycosis usually affects Caucasian patients, such as in the present report. We describe a form of rare geographic *Tinea nigra* with a speckled pattern.

The “salt and pepper” aspect should be taken into consideration when the mycosis is suspected.
